# Synchrotron X-ray powder diffraction validation of real-time in situ terahertz-Raman monitoring of resonant acoustic mixing cocrystallization

**DOI:** 10.1038/s42004-026-02072-w

**Published:** 2026-07-16

**Authors:** Cameron B. Lennox, Jogirdas Vainauskas, Joseph M. Marrett, Jean-Louis Do, Mihails Arhangelskis, Filip Topić, Michael Ferguson, Alexander Wahrhaftig-Lewis, Martin Etter, Andrew J. Morris, Tomislav Friščić, Tristan H. Borchers

**Affiliations:** 1 School of Chemistry, University of Birmingham Edgbaston, Birmingham, UK; 2 Department of Chemistry, McGill University, 801 Sherbrooke St. W., Montreal, Canada; 3 Department of Chemistry, Concordia University, 1455 Blvd. De Maisonneuve Ouest, Montreal, Canada; 4 Faculty of Chemistry, University of Warsaw, 1 Pasteura Street, Warsaw, Poland; 5 P02.1 Powder Diffraction and Total Scattering Beamline, Deutsches Elektronen-Synchrotron, Notkestraße 85, Hamburg, Germany; 6 School of Metallurgy and Materials, University of Birmingham, Edgbaston, Birmingham, UK; 7 Birmingham Centre for Mechanochemistry and Mechanical Processing, University of Birmingham, Edgbaston Birmingham, UK

**Keywords:** Reaction kinetics and dynamics, Halogen bonding, Raman spectroscopy

## Abstract

While synchrotron-based X-ray powder diffraction (XRPD) is often seen as the “gold standard” for real-time in situ monitoring of transformations of crystalline phases under mechanochemical conditions, an exciting opportunity to perform such studies using laboratory benchtop instrumentation is offered by terahertz-Raman (THz-Raman) spectroscopy, capable of detecting changes in lattice phonon bands. Here, we integrate synchrotron XRPD and THz-Raman into a tandem methodology that enabled the qualitative, as well as quantitative validation of the potential of THz-Raman spectroscopic monitoring of cocrystallization under Resonant Acoustic Mixing (RAM) conditions. By using halogen-bonded cocrystals that are difficult to differentiate using conventional Raman spectroscopy as model systems, this work reports strong agreement between the measured rate constants, as well as the ability to detect new crystalline phases by both THz-Raman spectroscopy and synchrotron XRPD. These results are an important first step in validating THz-Raman spectroscopy as a versatile, laboratory-based alternative to using synchrotron radiation for materials discovery and real-time in situ tracking of changes in materials structure, in both neat and liquid-assisted mechanically agitated processes.

**Figure:**
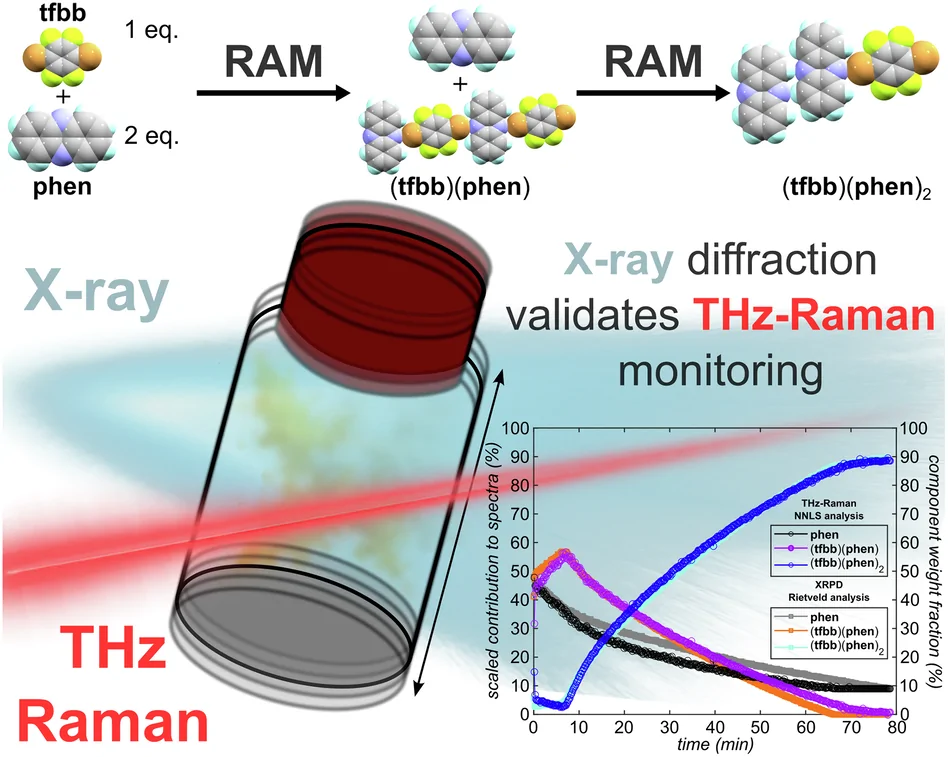

## Abstract (web summary)

Synchrotron-based X-ray powder diffraction is the benchmark for monitoring transformations of crystalline phases, but terahertz Raman spectroscopy offers a promising laboratory alternative. Here, the authors integrate synchrotron XRPD and THz-Raman spectroscopy under resonant acoustic mixing conditions, demonstrating strong agreement in rate constants and phase detection, validating THz-Raman as a versatile tool for real-time in situ tracking of changes in material structure.

## Introduction

Synchrotron X-ray powder diffraction (XRPD) is an ubiquitous tool for real-time in situ monitoring of solid-state transformations, particularly in the context of mechanochemical processes by grinding, milling, shearing, or other types of mechanical energy input^1–5^. Synchrotron XRPD can be seen as the “gold standard” for monitoring changes in extended crystalline structure, and is readily combined with Raman spectroscopy to detect changes in molecular structure^6,7^. While these complementary methods provide a unique and powerful toolkit for real-time monitoring of mechanochemical processes, the need for synchrotron radiation to follow changes in extended structure of materials represents an accessibility limitation^8–10^. Possible spectroscopic routes to probe changes in the long-range solid-state structure during mechanochemical transformations have been proposed, including solid-state nuclear magnetic resonance (ssNMR)^11,12^ and terahertz time-domain (THz-TD) spectroscopy^13–15^, but such approaches have generally required custom-built instrumentation or have been limited to ex situ analysis due to the requirement for additional sample preparation steps. In this context, the potential of Raman spectroscopy in the low-frequency/terahertz (THz) frequency region (typically between 0 and 200 cm^-1^) to follow changes in phonon modes in situ and in real time presents an exciting opportunity to expand the scope of benchtop studies of structural changes in mechanochemistry and mechanically-agitated systems in general^16,17^.

**Fig. 1:**
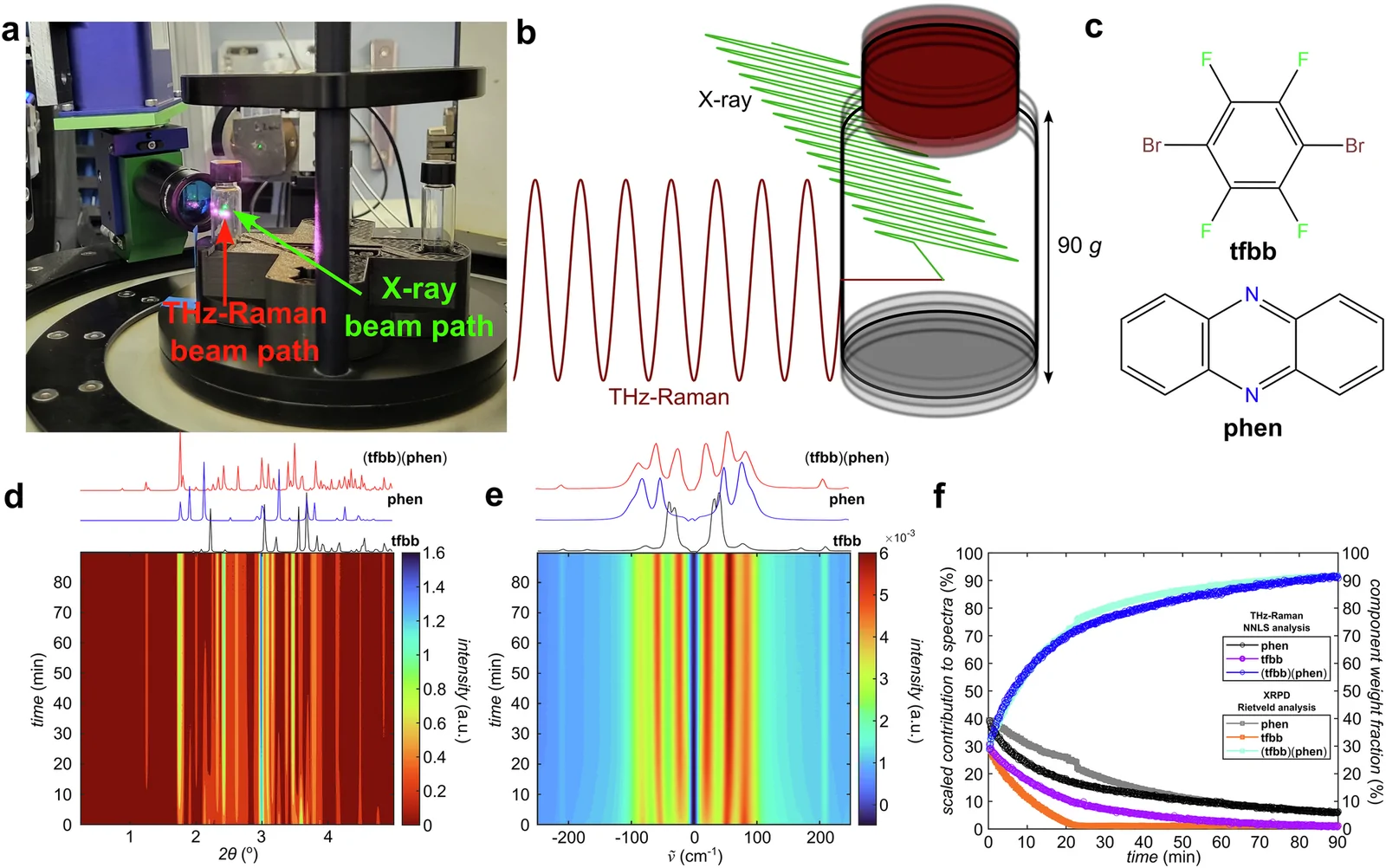
Tandem real-time monitoring of RAM cocrystallization. **a** The experimental setup highlighting the area of sampling, with the red and the green laser points showing the alignment of the THz-Raman probe, and of the synchrotron X-ray beam, respectively. **b** Schematic of the experiment design, illustrating the orientation of the oscillating sample with respect to the back-scattering Raman laser beam and the transmitted X-ray beam. **c** Molecular diagrams of **phen** and **tfbb**. **d** Time-resolved XRPD (*λ* = 0.20736 Å) waterfall plot of the reaction mixture of **tfbb** and **phen** in a 1:1 stoichiometric ratio during RAM at 90 *g* in the presence of EtOH at *η* = 1.0 μL·mg^-1^ (CCDC codes of reference patterns: ZZZAVJ01 (**tfbb**), PHENAZ12 (**phen**), JAYFUO (**tfbb**)(**phen**))^23,32,33^. **e** The corresponding time-resolved THz-Raman spectroscopy waterfall plot. **f** Comparison of the reaction profiles for the in situ experimental data from **d** and **e** based on Rietveld refinement of XRPD data and NNLS component fitting of THz-Raman spectroscopy data. The RAM experiment and monitoring were initiated ca. 5 minutes after the sample was prepared.

In order to evaluate this possibility, here we combine THz-Raman spectroscopy with synchrotron X-ray powder diffraction into a tandem method (Fig. 1a,b) that enables simultaneous diffraction^18^ and spectroscopy-based^19,20^ monitoring of materials structure transformations during cocrystal formation under Resonant Acoustic Mixing (RAM) in the acceleration range 0-100 *g* (*g* ~ 9.81 m·s^-2^), either neat, or in the presence of a liquid additive. By using as a model system the formation of halogen-bonded cocrystals that are not readily differentiated *via* conventional Raman spectroscopy, this tandem methodology enabled the quantitative benchmarking of THz-Raman spectroscopy against synchrotron XRPD for monitoring cocrystallization and cocrystal discovery^8^. The excellent quantitative agreement of spectroscopic data processed *via* a non-negative least-squares (NNLS) fitting algorithm^21^ with synchrotron XRPD data processed by Rietveld least-squares refinement^22^ now validates THz-Raman spectroscopy as a viable alternative for real-time monitoring of mechanically-agitated transformations of solids.

## Results and Discussion

The model system for tandem XRPD/THz-Raman monitoring was the formation of halogen-bonded cocrystals of phenazine (**phen**) as the halogen bond acceptor and 1,4-dibromotetrafluorobenzene (**tfbb**) (Fig. 1c) as the donor in a slurry system. Two stoichiometrically distinct cocrystal phases have previously been reported for this system, with compositions (**tfbb**)(**phen**) and (**tfbb**)(**phen**)_2_, consisting of supramolecular chains held *via* C-Br···N halogen bonds, or a combination of C-Br···N halogen bonds with C-H···N hydrogen bonds, respectively^23,24^. As the fingerprint region Raman spectra of (**tfbb**)(**phen**) and (**tfbb**)(**phen**)_2_ exhibit very little difference and are nearly indistinguishable from the linear combination of the spectra of the solid reactants (Supplementary Fig. 5-7), real-time monitoring of cocrystallization in this system by conventional Raman spectroscopy^17^ (>200 cm^-1^) would be difficult and would normally necessitate the use of synchrotron XRPD.

The X-ray beam (*λ* = 0.20736 Å, beam size = 1 × 1 mm²) was aligned to pass through the center of the reaction vial. While the 785 nm THz-Raman probe with a 30 mm non-contact objective was aligned *ca*. 45 ° with respect to the X-ray beam, focused on the center of the vial and collecting backscattered light (Fig. 1a-b, Supplementary Fig. 1-2, 4). In both cases, each scan comprised 10 seconds of total acquisition time (*i.e*., number of accumulations × integration time), allowing for the direct comparison of the XRPD and THz-Raman data.

The initial experiment involved mixing of an equimolar mixture of **phen** and **tfbb** (1 mmol scale, total weight 488 mg) by RAM at an acceleration of 90 *g*, along with a small amount of ethanol (EtOH, *η* = 1.0 μL·mg^-1^, 488 μL) as the liquid additive. Due to limitations in the experimental setup, there was an approximate five-minute delay between sample preparation and the start of the measurement, during which time the cocrystallization reaction had commenced, evident by both XRPD and THz-Raman data (Fig. 1d-f). Analysis of the in situ XRPD patterns revealed near-complete formation of the anticipated (**tfbb**)(**phen**) cocrystal within 90 minutes of mixing (Fig. 1d-f, Supplementary Fig. 8), evident by the disappearance of characteristic Bragg reflections corresponding to **phen** (*2θ* = 1.90 °), and the appearance of reflections corresponding to (**tfbb**)(**phen**) (*2θ* = 2.70 °) (Fig. 1d). While the reaction mixture appeared to be a slurry, the signals of **tfbb** in both THz-Raman and XRPD data were found to disappear before those of **phen**. This observation suggests that the process takes place *via* dissolution of **tfbb** in the liquid additive, with (**tfbb**)(**phen**) subsequently forming *via* a solution-mediated phase transformation (SMPT)^25–27^.

A similar trend was observed upon analysis of the in situ THz-Raman spectra (Fig. 1e), with the formation of (**tfbb**)(**phen**) apparent by the emergence of THz-Raman bands at 20 cm^-1^, 79 cm^-1^, 88 cm^-1^, and 208 cm^-1^, all of which continued to increase in intensity over the course of the reaction, coinciding with the disappearance of characteristic bands corresponding to **phen** (51 cm^-1^ to 57 cm^-1^) (Fig. 1d-f, Supplementary Fig. 8). This change in energy of phonon bands is indicative of a change to the crystalline environment of both **phen** and **tfbb**, as is expected for their incorporation into the target (**tfbb**)(**phen**) cocrystal.

To quantify the level of agreement between synchrotron XRPD and THz-Raman for real-time in situ monitoring, the corresponding reaction profiles (Fig. 1f, Supplementary Fig. 8) were fit using kinetic models. As the THz-Raman and XRPD reaction profiles of the formation of (**tfbb**)(**phen**) appeared to follow a non-linear rate, we have selected to fit the decay of **phen** to a single exponential rate law. The extent of reaction progress was determined by following the normalized integrations associated with the **phen** signal at 77 cm^-1^ and the **phen** X-ray reflection at *2θ* = 1.90 ° for THz-Raman and XRPD methods, respectively (Supplementary Fig. 8), over the course of the experiment. The decay of **phen** was found to fit a single exponential rate law, with good agreement between rate constants from integrated THz-Raman, *k*_THz_ 3.06(4) × 10^-2 ^min^-1^, and XRPD, *k*_XRPD_ 3.22(3) × 10^-2 ^min^-1^, data (Supplementary Figs. 9, 10). Rather than treating the data with simple signal integration, which might be affected by the presence of multiple overlapping bands in the case of THz-Raman spectroscopy, and/or preferred orientation effects for synchrotron XRPD, further analysis was conducted *via* NNLS fitting (*k*_THz_) and Rietveld refinement (*k*_XRPD_), respectively (Fig. 1f). This approach similarly demonstrated strong agreement between the two methods, with *k*_THz_ = 3.29(4) × 10^-2 ^min^-1^, and *k*_XRPD_ = 3.84(4) × 10^-2 ^min^-1^ (Supplementary Figs. 11, 12). It is important to note that the rate constants derived from both monitoring methods here serve only as a means of benchmarking the two methods and are not intended to inform upon the reaction mechanism.

To explore the mechanistic aspects of (**tfbb**)(**phen**) cocrystallization, we attempted to fit the experimentally monitored growth of the product phase to several Avrami kinetic models (A2, A3, and A4)^28–31^. Fitting each model to both the NNLS and Rietveld refinement datasets produced similar kinetic trends: in all cases, the cocrystal kinetics did not exhibit a single, continuous linear region. Instead, multiple approximately linear segments were observed, separated by non-linear intervals. The initial linear regime from approximately 1–20 min is associated with a faster growth rate (Supplementary Fig. 13**)**. The growth rate lowers in the second linear regime, spanning 25–60 min (Supplementary Fig. 14**)**. These results indicate that the overall transformation slows with time and suggest that cocrystal growth does not adhere to a single ideal Avrami–Erofe'ev mechanism^28–31^, but instead proceeds through at least two distinct kinetic regimes.

**Fig. 2:**
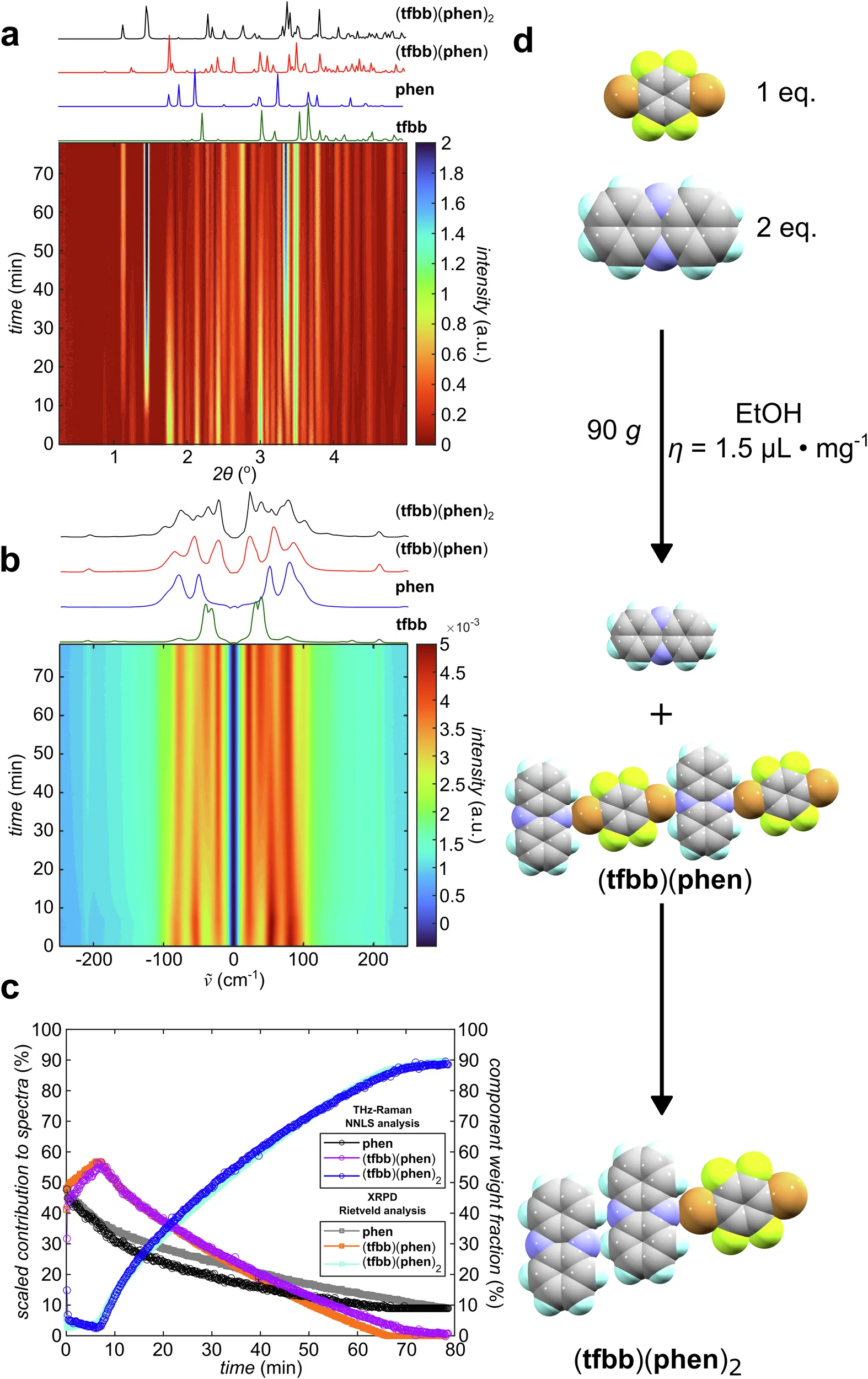
Real-time in situ monitoring of the effect of liquid additive on the cocrystallization of phen and tfbb to form (phen)(tfbb)_2_. **a** Time-resolved XRPD waterfall plot of the reaction of **tfbb** and **phen** in a 1:2 respective stoichiometric ratio, under RAM conditions at 90 *g* in presence of EtOH additive (*η* = 1.5 μL·mg^-1^) (*λ* = 0.20736 Å, CCDC codes of reference patterns: ZZZAVJ01 (**tfbb**), PHENAZ12 (**phen**), JAYFUO (**tfbb**)(**phen**), VOMGUA (**tfbb**)(**phen**)_2_)^23,24,32,33^. **b** Simultaneously collected corresponding time-resolved THz-Raman spectroscopy waterfall plot. **c **Comparison of the resulting reaction profiles based on NNLS processing of spectroscopic data and Rietveld refinement of XRPD data. **d** Schematic of the reaction, established by in situ monitoring. The RAM experiment and monitoring were initiated ca. 5 min after the sample was prepared.

Next, we explored the formation of the (**tfbb**)(**phen**)_2_ cocrystal using tandem XRPD/THz-Raman monitoring. A 1:2 stoichiometric mixture of **tfbb** and **phen**, along with EtOH (*η* = 1.5 μL·mg^-1^, 501 μL) upon RAM at 90 *g* rapidly generated (**tfbb**)(**phen**) (Fig. 2a-c), as seen from simultaneous increases in intensity of a characteristic Bragg reflection at *2θ* = 2.43° and a THz-Raman band at 22 cm^-1^. Further RAM led to the target (**tfbb**)(**phen**)_2_ cocrystal, identified by a distinctive Bragg reflection at *2θ* = 1.45 ° and a THz-Raman band centered at 40 cm^-1^. The appearance of (**tfbb**)(**phen**)_2_ was accompanied by a decay in the THz-Raman and XRPD signals of both (**tfbb**)(**phen**) and **phen** (Fig. 2a-d). The signals of **tfbb** were again difficult to account for, suggesting early dissolution. Analysis of the reaction profile for the formation of (**tfbb**)(**phen**)_2_ and the decay of **phen** and (**tfbb**)(**phen**) signals (Supplementary Figs. 15, 16) by NNLS fitting of the THz-Raman data and Rietveld refinement of the XRPD data gave remarkably consistent results between the two techniques. Treating the decay of **phen** and (**tfbb**)(**phen**) as a pseudo first-order process resulted in a rate constant of *k*_*THz*_ = 3.08(4) x 10^-2 ^min^-1^ and 3.32(3) x 10^-2 ^min^-1^, respectively for THz-Raman data (Supplementary Fig. 15), with similar values obtained by Rietveld analysis of XRPD data, *k*_XRPD_ = 3.14(3) × 10^-2 ^min^-1^ and 3.67(6) × 10^-2 ^min^-1^, respectively (Supplementary Fig. 16).

**Fig. 3:**
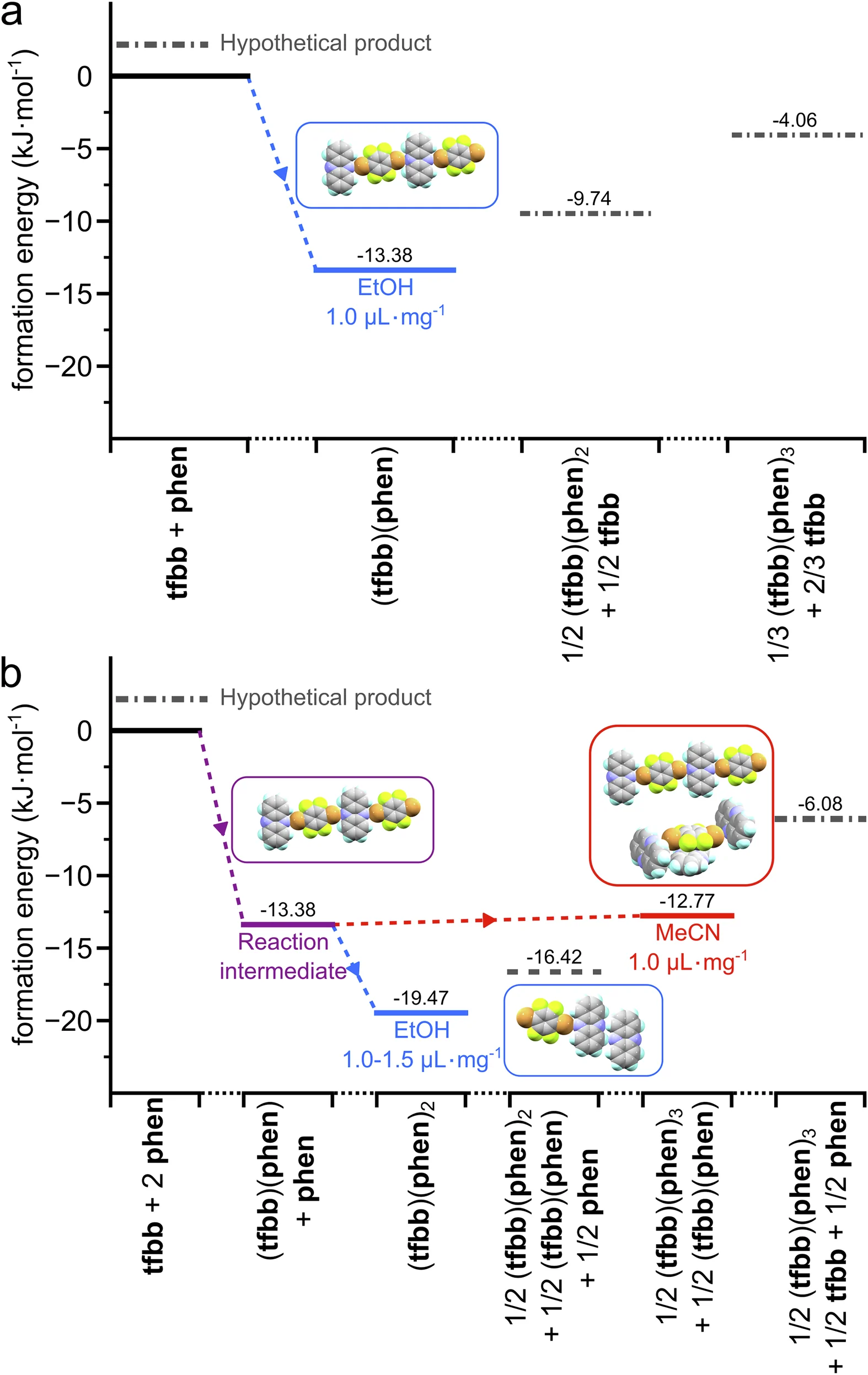
Energy landscape for the known cocrystals of tfbb with phen, based om periodic DFT calculations. **a** The energy of formation landscape for the reaction of **phen** and **tfbb** in a 1:1 stoichiometric ratio and **b** for the reaction in a respective 2:1 stoichiometric ratio, with different reaction pathways observed in presence of EtOH and MeCN shown in blue and red, respectively.

The sequence of solid phases observed by XRPD and THz-Raman monitoring is consistent with the relative stabilities of (**tfbb**)(**phen**), (**tfbb**)(**phen**)_2_, **phen** and **tfbb** solids (CCDC codes: JAYFUO, VOMGUA, PHENAZ12, and ZZZAVJ01 respectively, with the herein re-determined crystal structure for (**tfbb**)(**phen**) included as Supplementary Data 1)^23,32,33^, determined by periodic density-functional theory (DFT) calculations in CASTEP, v22.11^34^. The energies of the structures of each cocrystal, as well as **phen** and **tfbb** starting solids, were calculated using geometry optimizations with Perdew–Burke–Ernzerhof (PBE)^35^ functional combined with Grimme D3(0) dispersion correction^36^. The energies of the individual crystal structures were then used to compute the reaction energies, revealing that the formation of (**tfbb**)(**phen**) from a 1:1 mixture of **phen** and **tfbb** is thermodynamically favourable by -13.38 kJ·mol^-1^, with the formation of (**tfbb**)(**phen**)_2_ being less thermodynamically favoured (Fig. 3a). However, for a 1:2 stoichiometric mixture of **tfbb** and **phen**, the combined energy of formation for (**tfbb**)(**phen**) and **phen** was calculated to be -13.38·kJ mol^-1^, while the energy of formation for (**tfbb**)(**phen**)_2_ was calculated as -19.47 kJ·mol^-1^ (Fig. 3b). The calculated energies of formation are consistent with the experimental observation that (**tfbb**)(**phen**) forms when equimolar quantities of reactants are mixed, and suggests that the appearance of (**tfbb**)(**phen**) as an intermediate in the formation of (**tfbb**)(**phen**)_2_ follows the Ostwald rule of stages ^37–39^.

Next, the effect of the amount of liquid additive on the formation of (**tfbb**)(**phen**)_2_ (Supplementary Fig. 17-19) was explored by varying *η* from 0 μL·mg^-1^ (neat conditions) to 1.5 μL·mg^-1^ ^40,41^. Upon decreasing the amount of EtOH from *η* = 1.5 μL·mg^-1^, we observed a reduction in the rate of formation for both the (**tfbb**)(**phen**) intermediate phase, and the (**tfbb**)(**phen**)_2_ product phase. This change in reactivity might indicate reduced mixing efficiency in the presence of a smaller quantity of a liquid additive, most evident for *η*-values between 0.25 μL·mg^-1^ and 0.50 μL·mg^-1^.

The low *η-*value experiments were characterized by a significant variation in the intensity of the Raman signal (Supplementary Fig. 18), which was ascribed to poor mixing and was evident by visual monitoring, with the final product (**tfbb**)(**phen**)_2_ not observed by XRPD or THz-Raman spectroscopy even after 80 mins of RAM at 90 *g*. Interestingly, the neat reaction (*η* = 0 μL·mg^-1^) appeared to mix readily, leading to partial conversion to (**tfbb**)(**phen**) (calc. 19% weight fraction *via* Rietveld analysis), observed in both THz-Raman and XRPD data (Supplementary Fig. 19, 20) after 80 min of RAM at 90 *g*. Notably, under neat reaction conditions, the reaction profiles determined by THz-Raman and synchrotron XRPD were in close agreement (Supplementary Fig. 21).

**Fig. 4:**
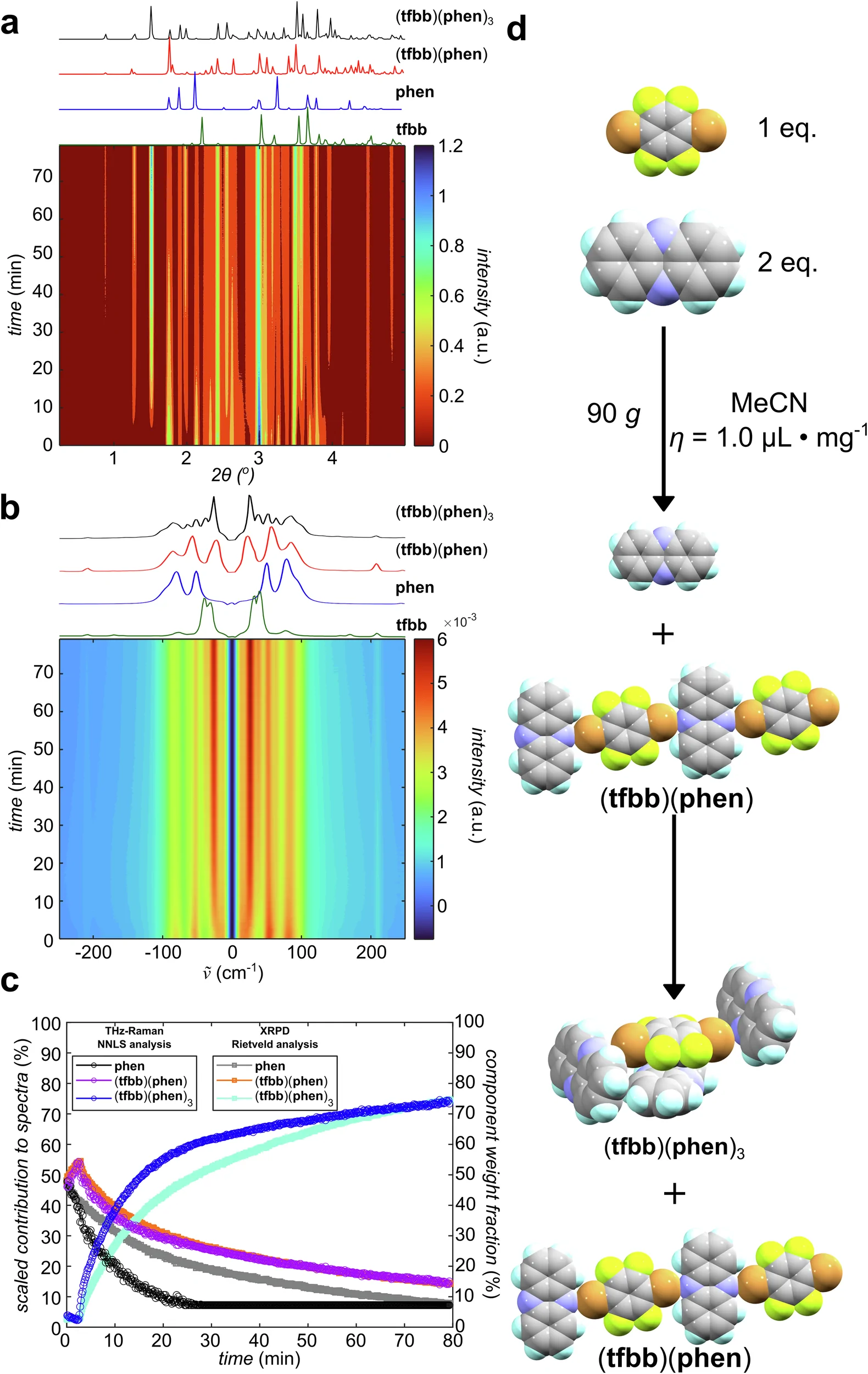
Real-time in situ monitoring of the effect of liquid additive on the cocrystallization of tfbb and phen in a 1:2 stoichiometric ratio. **a** The time-resolved XRPD waterfall plot of the reaction of **tfbb** and **phen** in a 1:2 stoichiometric ratio under RAM conditions at 90 *g*, in the presence of MeCN (*η* = 1.0 μL·mg^-1^, *λ* = 0.20736 Å, CCDC codes of reference patterns: ZZZAVJ01 (**tfbb**), PHENAZ12 (**phen**), JAYFUO (**tfbb**)(**phen**))^23,32,33^. **b** The corresponding time-resolved THz-Raman spectroscopy waterfall plot. **c** The resulting reaction profiles based on NNLS analysis and Rietveld refinement of THz-Raman spectroscopy and XRPD data, respectively. **d** Schematic representation of the reaction, based on in situ monitoring. The RAM experiment and monitoring were initiated ca. 5 minutes after the sample was prepared.

As the choice of liquid additive can influence cocrystallization under liquid-assisted conditions^42–45^, we also explored the effect of adding acetonitrile (MeCN, *η* = 1.0 μL·mg^-1^, 636.4 μL) to a 1:2 stoichiometric mixture of **tfbb** and **phen** (1 mmol scale, 636.4 mg). Upon RAM at 90 *g*, the reaction produced (**tfbb**)(**phen**) within the first two minutes of mixing, along with a small amount of a new crystalline phase, distinct from all known phases. The quantity of the new phase, identifiable through new Bragg reflections at 1.51° *2θ*, 2.57° *2θ*, and 3.53° *2θ*, and THz-Raman bands at 24 cm^-1^, 51 cm^-1^, and 84 cm^-1^ (Fig. 4a-d, Supplementary Fig. 22), increased upon further mixing, while the amount of (**tfbb**)(**phen**) decreased. The novel phase was subsequently isolated by fast recrystallization of the material from hot CHCl_3_, yielding single crystals suitable for X-ray structural analysis. Crystallographic analysis demonstrated a previously not reported cocrystal of composition (**tfbb**)(**phen**)_3_, isostructural to the previously reported 1,4-diiodotetrafluorobenzene (**tfib**) analogue^17^ and comprised of alternating layers of **tfbb** and **phen** stacked along the crystallographic *b*-axis (Fig. 4d, Table S1, see also Supplementary Data 2 for crystallographic data on the **(tfbb)(phen)**_**3**_ cocrystal). In the structure, each **tfbb** unit is engaged in two Br ∙ ∙∙N halogen bonds to adjacent **phen** units (Br ∙ ∙∙N distance = 3.220(1) Å), resulting in discrete **phen**-**tfbb**-**phen** trimers. The trimers link further into extended one-dimensional chains *via* **phen**-**phen** π-π stacking interactions.

Overall, analysis of the real-time data revealed that THz-Raman and synchrotron XRPD are equally effective in identifying the emergence of new phases during cocrystallization. Additionally, in situ THz-Raman and XRPD analysis revealed that, while (**tfbb**)(**phen**) is formed as an intermediate phase in the presence of either liquid additive (MeCN, EtOH), the targeted (**tfbb**)(**phen**)_2_ cocrystal is not accessible by MeCN (Fig. 4a-d).

The rate of formation of (**tfbb**)(**phen**)_3_ in the presence of MeCN, determined by THz-Raman monitoring, appeared to be higher than what is observed by XRPD monitoring (Fig. 4c). A potential explanation for such a difference is that THz-Raman spectroscopy is limited largely to probing backscattered light from material located at or near the reaction vessel surface, while XRPD integrates the X-ray scattering throughout the entire reaction vial. Such an explanation would imply that (**tfbb**)(**phen**)_3_ is not homogeneously distributed in the reaction mixture, but is more abundant at, and perhaps adheres more strongly to, the vial surface. In such a case, analyzing reaction kinetics *via* a surface-based measurement such as Raman scattering would not be expected to yield the same outcome as a through-volume X-ray diffraction study.

Replacing MeCN with CHCl_3_ (*η* = 1.0 μL·mg^-1^ , 636.4 μL) as the liquid additive led to the rapid appearance of (**tfbb**)(**phen**)_3_, with characteristic Bragg reflections and Raman bands corresponding to the product already detectable at the experiment start (Supplementary Fig. 23).

The difference in reactivity observed for the 1:2 stoichiometric mixture of **tfbb** and **phen** when using different liquid additives was addressed by periodic DFT calculations. From the relative energy of formations (Fig. 3b), and assuming behavior consistent with the Ostwald rule of stages^37–39^, a 1:2 mixture of **tfbb** and **phen** would be expected to first form the (**tfbb**)(**phen**) intermediate, regardless of the choice of the liquid additive. At that point, two diverging reaction pathways are available to the system. In the presence of EtOH, the reaction proceeds towards the most stable material for that stoichiometric composition, *i.e*., the (**tfbb**)(**phen**)_2_ cocrystal. However, in the presence of MeCN, the reaction proceeds to initially form (**tfbb**)(**phen**), before (**tfbb**)(**phen**)_3_ progressively forms until all available **phen** is consumed. Moreover, similar behavior was evident from in situ THz-Raman monitoring of a RAM experiment with MeCN liquid additive (*η* = 1.0 μL·mg^-1^, 90 *g*) starting from separately pre-synthesized (**tfbb**)(**phen**)_2_. Spectral analysis revealed rapid decay of bands of (**tfbb**)(**phen**)_2_, coinciding with an increase in intensity of THz-Raman bands corresponding to (**tfbb**)(**phen**)_3_ (Supplementary Fig. 24). After ca. 10 minutes, the reaction appeared to stagnate and signals corresponding to (**tfbb**)(**phen**)_3_ and residual (**tfbb**)(**phen**) remained largely invariant. The calculated energies of formation for a mixture of (**tfbb**)(**phen**) with either **phen**, or (**tfbb**)(**phen**)_3_ are -13.38 kJ·mol^-1^ and -12.77 kJ·mol^-1^, respectively. These values indicate that the two mixtures would be effectively equienergetic, within the expected accuracy of periodic DFT calculations^46,47^. Both energies of formation are, however, less favorable than the one calculated for the (**tfbb**)(**phen**)_2_ cocrystal (-19.47 kJ·mol^-1^). Overall, the experimental and theoretical data suggest that the formation of a mixture with (**tfbb**)(**phen**)_3_, rather than (**tfbb**)(**phen**)_2_, in the presence of MeCN is not the result of the relative thermodynamic stability of the pure solid phases, but is instead likely affected by the distinct solvation environment created by MeCN and EtOH ^48^.

Finally, we explored RAM of a 1:1 stoichiometric mixture of pre-synthesized (**tfbb**)(**phen**)_3_ and (**tfbb**)(**phen**) cocrystals in the presence of EtOH (*η* = 1.5 μL·mg^-1^). After 80 min at 90 *g*, no significant changes were observable in the THz-Raman signal (Supplementary Fig. 25), indicating that the mixture of (**tfbb**)(**phen**)_3_ and (**tfbb**)(**phen**) persists throughout the process. This outcome is different than what is observed in a direct reaction of **tfbb** and **phen** in a 1:2 stoichiometric ratio in the presence of EtOH, which first generates (**tfbb**)(**phen**), than subsequently transforms into (**tfbb**)(**phen**)_2_. The described results indicate (**tfbb**)(**phen**)_3_ is a kinetically persistent phase that does not readily transform under herein explored RAM conditions ^18^.

In summary, the herein presented tandem real-time in situ monitoring methodology has demonstrated how THz-Raman spectroscopy can be used along with synchrotron-based XRPD to follow transformations in neat and liquid-assisted systems, providing simultaneous and complementary information on crystal structure and intermolecular vibrational modes. The presented tandem measurements, conducted on systems that range from neat, solventless reactions to slurries, demonstrate that THz-Raman spectroscopy can indeed qualitatively and quantitatively perform comparably to synchrotron XRPD for both following the kinetics of cocrystallization, as well as for observation of novel crystalline phases, including for systems that would be difficult to distinguish by conventional Raman monitoring. In that context, this work also notes a potential limitation when comparing THz-Raman and XRPD data. Specifically, we find that when reaction mixtures are not sufficiently homogeneous, reaction rates are detected differently by surface-based Raman and through-volume X-ray diffraction techniques. By validating real-time in situ THz-Raman spectroscopy against the synchrotron XRPD for monitoring mechanically-promoted transformations of molecular solids, this work provides an opportunity to further enrich the toolkit of laboratory-based methods for monitoring mechanically-driven transformations^19,20,49–51^. Further work on validating the results of THz-Raman against synchrotron XRPD monitoring in other, chemically different systems is ongoing.

## Methods

### Materials

All reagents and solvents were obtained from commercial sources and were used without further purification. Phenazine was obtained from Sigma Aldrich, 1,4-dibromotetrafluorobenzene was obtained from Fluorochem, ethanol and acetonitrile were purchased from Fisher Scientific.

### THz-Raman Monitoring

The in situ terahertz-Raman (THz-Raman) monitoring experiments were performed using a Coherent THz-Raman system equipped with a 785 nm clean-line laser, a TR-probe, and a non-contact optic accessory including a 30 mm focal length achromatic doublet lens. The probe was aligned such that the focal length of the lens was at the center of the vial. Data collection was continuous throughout the entire experiment, with total acquisition time of 10 s per spectrum. Spectra were acquired in the frequency range of -500–1800 cm^-1^, with the laser power set to 30% of the available 300 mW power, producing 90 mW of laser power at the base of the laser module prior to entering the THz-Raman probe. Temperature of the hutch remained constant *ca*. 23.0 ± 0.1 °C, with any heating due to the laser probe likely to be compensated by rapid vial motion.

### X-ray diffraction monitoring

The in situ X-ray powder diffraction (XRPD) experiments were performed at the Deutsches Elektronensynchrotron (DESY), Hamburg, Germany at beamline P02.1 using a ca. 1 mm^2^ unfocused and collimated X-ray beam. The beamline is equipped with a Varex XRD4343 CT 2D area detector. The RAM was placed such that the X-ray beam passed directly through the centre of the vial. The wavelength (*λ*) was determined, using a NIST NSR 660c (LaB6) standard and measuring two different detector positions, to be 0.20736 Å (59.80 keV) for all in situ XRPD measurements herein described. All 2D XRPD patterns were integrated using either Fit2D or Dioptas^52,53^. Data acquisition was continuous throughout the experiment with a total acquisition time of 10 s per scan.

### Raman spectra analysis

Reaction profile estimates for in situ datasets were created using MATLAB2023a. The original dataset was baseline corrected using the Sonneveld and Visser algorithm^54^, truncated from -250 to 250 cm^-1^, and the data was normalized by area under the curve Eq. 1, using the trapezoid rule.

1 $${Normalized} \, {intensity}=\frac{{Intensity}}{\int {Intensity}}$$

The data was then analyzed using a non-negative linear least squares (NNLS) fitting algorithm (“lsqnonneg” in MATLAB)^21^, wherein each in situ measured spectra is fit as a sum of the normalised component spectra. Processing was performed analogous to previous reported work^55^. The resultant reaction progress curves where then scaled to fit the percentage weight fraction of the Rietveld analysis of the XRPD data.

### Powder X-ray Diffraction pattern analysis

Analysis of X-ray diffraction powder patterns and reaction profile estimates (integrated intensities) for in situ datasets were created in MATLAB2023a. The raw data was truncated from 0.25^o^ *2θ* to 5^o^ *2θ*, after which the raw data was treated through background subtraction *via* built in MATLAB function “msbackadj” (Supplementary Fig. 3), followed by picking reflections to integrate based on intensity and relative position of the reflection to other species. Additionally, the data was normalized by by area under the curve Eq. 1, using the trapezoid rule.

### Rietveld refinement of in situ XRPD data

Rietveld refinement was performed on the raw diffraction data using the code TOPAS Academic 7^22,56^. Before performing the sequential refinement of in situ data, unit cell parameters of individual crystal structures of starting materials and cocrystals were refined against room-temperature XRPD data, in order to account for the unit cell expansion of the structures, for which single crystal X-ray single crystal diffraction measurements were performed at low temperature *ca*. 100–253 K. The unit cell parameters obtained during room temperature Rietveld refinement, were subsequently kept fixed during the analysis of the in situ data.

The sequential Rietveld refinement^22^ of synchrotron in situ XRPD data was performed sequentially, starting from the scan corresponding to the start of the reaction monitoring, and proceeding through all the scans in the order of data acquisition. The converged refinement parameters for each scan were used as a starting point for the refinement of the next scan. The background was described by a 24-term Chebyshev polynomial, diffraction peak shapes were modelled with a pseudo-Voigt function. The composition of the reaction mixture was determined by refining the scale factors corresponding to the individual crystal phases, and subsequently calculating the corresponding weight fractions of the materials in the reaction mixture, examples are given of selected refinements in the SI (Supplementary Figs. 20, 26 – 28). The resulting weight fractions were then used to plot the kinetic profiles for comparison with the results of THz-Raman NNLS analysis.

### Single crystal X-ray diffraction

Single crystal X-ray diffraction data were collected on a Bruker D8 Venture dual-source diffractometer equipped with a PHOTON II detector and an Oxford Cryostream 800 cooling system, using mirror-monochromatized Mo*K*_α_ (*λ* = 0.71073 Å) radiation from microfocus sources. Data was collected in a series of *φ*- and *ω-*scans. APEX3 software was used for data collection, integration, and reduction^57^. Semi-empirical absorption correction was applied using SADABS-2016/2^58^. The structures were solved by dual-space iterative methods using SHELXT-2014/5^59^ or 2018/2 and refined by full-matrix least-squares on *F*^*2*^ using all data with SHELXL-2018/3^60^ within OLEX2 and WinGX^61^ packages. All non-hydrogen atoms were refined anisotropically. Hydrogen atoms were placed in calculated positions and treated as riding on the parent carbon atoms with *U*_iso_(H) = 1.2 *U*_eq_(C). Extinction correction was applied as implemented in SHELXL-2018/3.

CCDC 2474390- 2474391 contain the supplementary crystallographic data for this paper. The data can be obtained free of charge from the Cambridge Crystallographic Data Centre *via* www.ccdc.cam.ac.uk/structures.

### Theoretical methods

Periodic DFT calculations were performed using the plane-wave periodic DFT code CASTEP 22.11^34^. The crystal structure information (CIF format) for all herein explored cocrystals, as well as **phen** and **tfbb** starting materials, were converted to the CASTEP input format using the cif2cell code^62^. Calculations were performed with the PBE functional^29^, combined with the Grimme D3(0) semiempirical dispersion correction^30^. The plane-wave basis set was truncated at the 1200 eV cutoff, using the built-in norm-conserving pseudopotentials. The 1st electronic Brillouin zone was sampled with a Monkhorst-Pack *k*-point grid^63^ with a 2π × 0.04 Å^-1^ *k*-point spacing. These settings were used in all steps of the periodic DFT calculations.

### Resonant Acoustic Mixing experiments

Experiments were carried out using a LabRAM II (Resodyn) instrument, with commercial (FisherBrand #11523532) 5 mL borosilicate glass vials used as reactor vessels. In a typical experiment, one mmol of halogen bond donor, 1,4-tetrafluorobromobenzene and the required stoichiometric equivalent for the halogen bond acceptor phenazine placed on the resonant acoustic mixer (RAM), and mixed for 30 to 90 min with a liquid additives (EtOH, MeCN, or CHCl_3_) at *η*-values from 0 to 1.5 μL·mg^-1^ or neat *η* = 0 μL·mg^-1^. Acceleration for all samples was set to 90 *g*. Reference samples were prepared following the same procedure (mixing for 30 to 90 min) with phase composition confirmed by XRPD.

A RAM sample holder was designed using the TinkerCAD 3D modelling software package and sliced with ideaMaker, before being printed using a Raise3D pro2 printer with a 0.4 mm brass nozzle with PolyMaker PolyTerra© PLA filament. 3D printing files are supplied as components of the supporting information. For synchrotron XRPD experiments, an additional beam stop was added to the sample holder (Supplementary Fig. 4).

### Materials availability

All materials used in this study are either commercially available or can be prepared using procedures indicated in Supporting Information.

## Supplementary information

Supplementary Information

Description of Additional Supplementary Files

Supplementary Data 1

Supplementary Data 2

## Data Availability

The THz-Raman and synchrotron PXRD datasets for this study are available from Zenodo (https://doi.org/10.5281/zenodo.20842934) or from the corresponding authors on reasonable request. Crystallographic data in CIF format and CheckCIF reports for (**tfbb**)(**phen**) and (**tfbb**)(**phen**)_3_ are provided in Supporting Information. Specifically, the crystallographic files for structures of (**tfbb**)(**phen**) and (**tfbb**)(**phen**)_3_ are provided as Supplementary Data 1 and Supplementary Data 2, respectively. CCDC 2474390 and CCDC 2474391 contain the supplementary crystallographic data for this paper. This data can be obtained free of charge from the Cambridge Crystallographic Data Centre *via* www.ccdc.cam.ac.uk/structures.
