# Supplementary material for: Synchrotron X-ray powder diffraction validation of real-time in situ terahertz-Raman monitoring of resonant acoustic mixing cocrystallization
Source: Commun Chem. 2026 Jul 16;9:308. doi: 10.1038/s42004-026-02072-w (PMC13614948; doi:10.1038/s42004-026-02072-w)
Supplement: Supplementary file 1 [file 42004_2026_2072_MOESM2_ESM.pdf]

Description of Additional Supplementary Files:

File name: Supplementary Data 1.cif

Description: Crystallographic data in CIF format for the (**tfbb**)(**phen**) cocrystal at 253 K.

File name: Supplementary Data 2.cif

Description: Crystallographic data in CIF format for the **(tfbb)(phen)**$_3$ cocrystal at 253 K.
