# Supplementary material for: Synchrotron X-ray powder diffraction validation of real-time in situ terahertz-Raman monitoring of resonant acoustic mixing cocrystallization
Source: Commun Chem. 2026 Jul 16;9:308. doi: 10.1038/s42004-026-02072-w (PMC13614948; doi:10.1038/s42004-026-02072-w)
Supplement: Supplementary file 2 [file 42004_2026_2072_MOESM1_ESM.docx]

**Supporting Information for:**

**Synchrotron X-ray powder diffraction validation of real-time in situ terahertz-Raman monitoring of resonant acoustic mixing cocrystallization**

Cameron B. Lennox, Jogirdas Vainauskas, Joseph M. Marrett, Jean-Louis Do, Mihails Arhangelskis, Filip Topić, Michael Ferguson, Alexander Wahrhaftig-Lewis, Martin Etter, Andrew J. Morris, Tomislav Friščić,* and Tristan H. Borchers*

**Table of contents**

**S1**	Experimental Details	**2-5**

**S1.1**	Experimental methods	**2**

**S1.2**	Powder X-ray diffraction pattern analysis	**3**

**S1.3**	Single crystal X-ray diffraction	**4**

**S1.4**	Resonant Acoustic Mixing synthesis	**5**

**S2**	Cocrystalization of **tfbb** and **phen** in a 1:1 ratio	**5-12**

**S3**	Cocrystalization of **tfbb** and **phen** in a 1:2 ratio	**13-18**

**S4**	Selected Rietveld refinements to determine reaction mixture	**19-21**

S1 **Experimental details**
   S1.1 **Experimental methods**

**Supplementary Figure 1.** Image of the experimental setup, highlighting that the beam-alignment green laser (red box) is positioned such that it passes through the center of the vial, representative of the path of the X-ray beam with respect to the Raman probe. Additionally, mounted sample holder (black), and the 5 mL glass sample vial are shown.

**Supplementary Figure 2.** Image of experimental setup, with the beam-alignment green laser passing through the center of the vial, indicating the path of the X-ray beam with respect to the Raman probe. Additionally, 3D sample holder (black), and the 5 mL glass sample vial are shown.

S1.2 **Powder X-ray diffraction pattern analysis**

**Supplementary Figure 3**. Example of background subtraction for a XRPD pattern, showing the calculated baseline from the estimated baseline points and how they fit to the original signal. For background modelling used for Rietveld analysis, see Methods section in the manuscript.

S1.3 **Single crystal X-ray diffraction**

CCDC 2474390- 2474391 contain the supplementary crystallographic data for this paper. The data can be obtained free of charge from the Cambridge Crystallographic Data Centre *via* [www.ccdc.cam.ac.uk/structures](http://www.ccdc.cam.ac.uk/structures).

**Table S1.** Crystallographic and structure refinement data for (**tfbb**)(**phen**) and (**tfbb**)(**phen**)_3_.

|  | (**tfbb**)(**phen**) | (**tfbb**)(**phen**)_3_ |
| --- | --- | --- |
| Empirical formula | C_18_H_8_Br_2_F_4_N_2_ | C_42_H_24_Br_2_F_4_N_6_ |
| Formula weight | 488.08 | 848.49 |
| *T* / K | 253.0(1) | 253.0(1) |
| Crystal system | triclinic | triclinic |
| Space group | *P*-1 | *P*-1 |
| *a* / Å | 6.2293(4) | 6.9182(6) |
| *b* / Å | 10.0847(7) | 9.4136(7) |
| *c* / Å | 14.4373(10) | 13.4738(10) |
| *α* / ° | 71.153(2) | 92.727(3) |
| *β* / ° | 81.289(3) | 92.886(3) |
| *γ* / ° | 85.348(2) | 99.402(3) |
| *Volume* / Å^3^ | 847.93(10) | 863.21(12) |
| *Z* | 2 | 1 |
| *ρ_calc_* / g cm^-3^ | 1.912 | 1.632 |
| *μ* / mm^‑1^ | 4.825 | 2.412 |
| *F*(000) | 472.0 | 424.0 |
| *Crystal size* / mm^3^ | 0.485 × 0.216 × 0.113 | 0.673 × 0.19 × 0.078 |
| Radiation | Mo*K*_α_ (λ = 0.71073 Å) | Mo*K*_α_ (λ = 0.71073 Å) |
| 2Θ range for data collection / ° | 5.95 to 61.404 | 5.478 to 61.128 |
| Index ranges | -8 ≤ h ≤ 8, -14 ≤ k ≤ 14, -20 ≤ l ≤ 20 | -9 ≤ h ≤ 9, -13 ≤ k ≤ 13, -19 ≤ l ≤ 19 |
| Reflections collected | 37650 | 30398 |
| Independent reflections | 5245 [*R_int_* = 0.0379, *R_sigma_* = 0.0259] | 4828 [*R_int_* = 0.0400, *R_sigma_* = 0.0249] |
| Data/restraints/parameters | 5245/0/236 | 4828/0/244 |
| Goodness-of-fit on *F*^2^ | 1.022 | 1.038 |
| Data completeness % | 99.4 | 91.3 |
| Final *R* indexes [*I ≥* 2*σ_I_*] | *R_1_* = 0.0350, w*R_2_* = 0.0706 | *R_1_* = 0.0311, w*R_2_* = 0.0776 |
| Final *R* indexes [all data] | *R_1_* = 0.0550, w*R_2_* = 0.0780 | *R_1_* = 0.0363, w*R_2_* = 0.0804 |
| Largest diff. peak/hole / e Å^-3^ | 0.82/-0.94 | 0.57/-0.51 |

S1.4 **Resonant Acoustic Mixing experiments**

**Supplementary Figure 4.** (from left to right) 3-dimensional models of the bottom (blue), top (magenta), and beam-stop components (green and brown) of the vial holder created for tandem THz-Raman spectroscopy and synchrotron XRPD monitoring on the LabRAM II reaction platform.

S2 **Cocrystallization of tfbb and phen in a 1:1 ratio under RAM conditions**

**Supplementary Figure 5.** Raman spectra from -250 cm^-1^ to 1800 cm^-1^ of previously reported **tfbb** and **phen** stoichiomorphs (**tfbb**)(**phen**) in red and (**tfbb**)(**phen**)_2_ in black. The differential plot between the two stoichiomorphs is displayed by hashed blue line, highlighting the large difference between the respective cocrystals in the THz-region and small differences between the respective cocrystals in the Raman fingerprint region.

**Supplementary Figure 6.** Full Raman spectra from -250 cm^-1^ to 1800 cm^-1^ of previously reported **tfbb** and **phen** cocrystal (**tfbb**)(**phen**) in blue, along with individual components **tfbb** (black) and **phen** (red). The differential plot between the (**tfbb**)(**phen**) and the sum of the components is displayed in cyan, highlighting the large difference between the cocrystal and components in the THz-region and small differences between the cocrystal and components in the Raman fingerprint region.

**Supplementary Figure 7.** Full Raman spectra from -250 cm^-1^ to 1800 cm^-1^ of the previously reported **tfbb** and **phen** cocrystal (**tfbb**)(**phen**)_2_ (blue) and individual components **tfbb** (black), **phen** (red). The difference plot between the (**tfbb**)(**phen**)**_2_** and the sum of the components is shown in cyan, highlighting the large difference between the cocrystal and components in the THz-region and small differences between the cocrystal and components in the Raman fingerprint region.

**Supplementary Figure 8.** Comparison of reaction progress for the formation of (**tfbb**)(**phen**) following mixing an equimolar mixture of **tfbb** and **phen** with EtOH at *η* = 1.0 μL·mg^-1^ (488 μL), based on integration of selected signals in XRPD (2.70 º *2θ*, orange) and THz-Raman (21 cm^-1^, blue) data.

**Supplementary Figure 9. a.** Normalized peak integration of a characteristic **phen** THz-Raman band at 77 cm^-1^, and **b.** the result of fitting the integrated data to a first order rate equation for the synthesis of (**tfbb**)(**phen**) by mixing an equimolar mixture of **tfbb** and **phen** at 90 *g* with EtOH at *η* = 1.0 μL·mg^-1^ (488 μL).

**Supplementary Figure 10. a.** Normalized peak integration of a characteristic **phen** Bragg reflection at 1.90 ^o^ *2θ*, and **b.** the result of fitting the integrated data to a first order rate equation for the synthesis of (**tfbb**)(**phen**) by mixing an equimolar mixture of **tfbb** and **phen** at 90 *g* with EtOH at *η* = 1.0 μL·mg^-1^ (488 μL).

**Supplementary Figure 11. a.** Relative contribution of **phen** based on NNLS analysis of the THz-Raman data and **b.** the result of fitting the NNLS data to a first order rate equation for the synthesis of (**tfbb**)(**phen**) by mixing an equimolar mixture of **tfbb** and **phen** at 90 *g* with EtOH at *η* = 1.0 μL·mg^-1^ (488 μL).

**Supplementary Figure 12. a.** Relative contribution of **phen** based on Rietveld refinement of XRPD data and **b.** result of fitting the Rietveld refinement data to a first-order rate equation for the synthesis of (**tfbb**)(**phen**) by mixing an equimolar mixture of **tfbb** and **phen** at 90 *g* with EtOH at *η* = 1.0 μL·mg^-1^ (488 μL).

**Supplementary Figure 13.** The initial growth kinetics (1 – 20 min) for the synthesis of (**tfbb**)(**phen**) by mixing an equimolar mixture of **tfbb** and **phen** at 90 *g* with EtOH at *η* = 1.0 μL·mg^-1^ (488 μL), fitted to a variety of Avrami models. Left) NNLS data fit to **a.** A2 Avrami model, **b.** A3 Avrami model, and **c.** A4 Avrami model. Right) XRPD data fit to **d.** A2 Avrami model, **e.** A3 Avrami model, and **f.** A4 Avrami model.

**Supplementary Figure 14.** The second area of linearity growth kinetics (25 – 60 min) for the synthesis of (**tfbb**)(**phen**) by mixing an equimolar mixture of **tfbb** and **phen** at 90 *g* with EtOH at *η* = 1.0 μL·mg^-1^ (488 μL), fitted to a variety of Avrami models. Left) NNLS data fit to **a.** A2 Avrami model, **b.** A3 Avrami model, and **c.** A4 Avrami model. Right) XRPD data fit to **d.** A2 Avrami model, **e.** A3 Avrami model, and **f.** A4 Avrami model.

S3 **Cocrystallization of tfbb and phen in a 1:2 ratio under RAM conditions**

**Supplementary Figure 15.** Analysis of the data for mixing of a 2:1 stoichiometry mixture of **tfbb** and **phen** at 90 *g* with EtOH at *η* = 1.5 μL·mg^-1^ (501 μL): **a.** The NNLS data for the component **phen** fitted to a first order rate equation. **b.** The NNLS data for the component (**tfbb**)(**phen**) fitted to a first order rate equation.

**Supplementary Figure 16.** Analysis of the data for mixing of a 2:1 stoichiometry mixture of **tfbb** and **phen** at 90 *g* with EtOH at *η* = 1.5 μL·mg^-1^ (501 μL): **a.** Rietveld refinement data for **phen** fitted to a first order rate equation. **b.** Rietveld refinement data for (**tfbb**)(**phen**) fitted to a first order rate equation.

**Supplementary Figure 17.** Relative contribution of (**tfbb**)(**phen**)_2_ to Raman spectra with EtOH added as liquid additive at *η*-values from 0.75 to 1.5 μL·mg^-1^ for **tfbb** and **phen** mixed in a 1:2 stoichiometric ratio at 90 *g*. The relative contribution of (**tfbb**)(**phen**)_2_ to each spectrum was determined by a NNLS fitting algorithm.

**Supplementary Figure 18.** THz-Raman spectroscopy time-resolved waterfall plot following the reaction of **tfbb** and **phen** mixed in a 1:2 stoichiometric ratio by RAM at 90 *g* for 60 minutes in the presence of EtOH at **a.** *η* = 0.25 μL·mg^-1^ and **b.** *η* = 0.50 μL·mg^-1^.

**Supplementary Figure 19. a.** Time-resolved waterfall plot from XRPD (*λ* = 0.20736 Å) and **b.** THz-Raman spectroscopy, following the neat reaction of **tfbb** and **phen** mixed in a 1:2 stoichiometric ratio by RAM at 90 *g* for 90 minutes.

**Supplementary Figure 20.** Rietveld Analysis of XRPD scan 462 (*λ* = 0.20736 Å) (80 mins of mixing), of neat reaction between **tfbb** and **phen** mixed in a 1:2 stoichiometric ratio by RAM at 90 *g*.

**Supplementary Figure 21.** Reaction profiles following the neat reaction of **tfbb** and **phen** mixed in a 1:2 stoichiometric ratio by RAM at 90 *g*. **a.** % Weight-fraction analysis of real-time XRPD data computed *via* Rietveld analysis. **b.** % Contribution of component to the spectra analysis of real-time THz-Raman data computed by NNLS fitting.

**Supplementary Figure 22.** Raman spectra from -250 cm^-1^ to 1800 cm^-1^ of the cocrystal (**tfbb**)(**phen**)_3_ and individual components **tfbb** and **phen**; blue (**tfbb**)(**phen**)_3_, black **tfbb**, and red **phen** . The differential between the (**tfbb**)(**phen**)_3_ and the sum of the components is seen in cyan, highlighting the large difference between the cocrystal and components in the THz-region and small differences between the cocrystal and components in the Raman fingerprint region.

**Supplementary Figure 23. a.** Time-resolved waterfall plot from XRPD (*λ* = 0.20736 Å) and **b.** THz-Raman spectroscopy following the reaction of **tfbb** and **phen** mixed in a 1:2 stoichiometric ratio in the presence of CHCl_3_ (*η* = 1.0 μL·mg^-1^) as liquid additive by RAM at 90 *g*.

**Supplementary Figure 24. a.** Time-resolved THz-Raman waterfall plot **b.** and reaction profile based on NNLS treatment of *in situ* THz-Raman spectra for monitoring of (**tfbb**)(**phen**)_2_ in the presence of MeCN as a liquid additive at *η* = 1.0 μL·mg^-1^ mixed by RAM at 90 *g*.

**Supplementary Figure 25. a.** Time-resolved THz-Raman waterfall plot and **b.** reaction profile based on NNLS treatment of *in situ* THz-Raman spectra for monitoring (**tfbb**)(**phen**)_3_ and (**tfbb**)(**phen**) in the presence of EtOH as a liquid additive at *η* = 1.5 μL·mg^-1^, mixed by RAM at 90 *g*.

S4 **Selected examples of Rietveld refinement used to determine composition of reaction mixture**

*

*

*

**Supplementary Figure 26.** Selected converged refinements for the XRPD real time *in situ* monitoring data for **phen** and **tfbb** in a 1:1 molar ratio, reacted in the presence of EtOH at *η* = 1.0 μL·mg^-1^ under mixing conditions of 90 *g*. There is a single reflection at 1.45 ^o^ *2θ* (highlighted by the * mark), which could not be assigned to any relevant phases. The reflection remains present throughout the entire reaction, with the intensity remaining constant throughout, suggesting this crystalline impurity is neither a reactant, reagent, product or an intermediate of the cocrystallization reaction studied.

**Supplementary Figure 27.** Selected converged refinements for XRPD real time *in situ* monitoring data of **tfbb** and **phen** in a 1:2 respective molar ratio, reacted in the presence of EtOH at *η* = 1.5 μL·mg^-1^ under mixing conditions of 90 *g*.

**Supplementary Figure 28.** Selected converged refinements for XRPD real time *in situ* monitoring data of **tfbb** and **phen** in a 1:2 respective molar ratio, reacted in the presence of MeCN at *η* = 1.0 μL·mg^-1^ under mixing conditions of 90 *g*.
